# Immobilization of a Fungal Fructosyltransferase onto Silica Gel and Glutaraldehyde-Functionalized Silica Gel for Biocatalytic Applications

**DOI:** 10.1021/acsomega.5c08802

**Published:** 2026-02-19

**Authors:** José Pedro Zanetti Prado, Ana Carolina Vieira, Alfredo Eduardo Maiorano, Sérgio Fernandes, Rodrigo Correa Basso, Sylma Carvalho Maestrelli, Cristiane Angélica Ottoni, Michelle da Cunha Abreu Xavier, Sergio Andres Villalba Morales, Rafael Firmani Perna

**Affiliations:** † Graduate Program in Chemical Engineering, Institute of Science and Technology, 74347 Federal University of Alfenas (UNIFAL-MG), Poços de Caldas 37715-400, MG, Brazil; ‡ Bionanomanufacturing Center, 154620 Institute for Technological Research (IPT-SP), São Paulo 05508-901, SP, Brazil; § Graduate Program in Materials Science and Engineering, Federal University of Alfenas (UNIFAL-MG), Poços de Caldas 37715-400, MG, Brazil; ∥ Institute of Biosciences, São Paulo State University (UNESP), São Vicente 11330-900, SP, Brazil; ⊥ Graduate Program in Food Science and Technology, 74385 Federal University of Tocantins (UFT), Palmas 77001-090, TO, Brazil

## Abstract

The immobilization of fructosyltransferase enzymes has been identified as an essential strategy for the production of fructooligosaccharides (FOS) in heterogeneous reaction systems. This study investigated the applicability of silica gel and silica gel functionalized with glutaraldehyde as porous inorganic supports for the immobilization of the extracellular fructosyltransferase from *Aspergillus oryzae* IPT-301, aiming to obtain active and stable heterogeneous biocatalysts for FOS production. The silicas were characterized using Fourier transform infrared (FTIR), scanning electron microscopy (SEM) and N_2_ physisorption. The thermal, operational, storage, and pH stability, as well as the kinetic profiles of the biocatalysts, were evaluated. The functionalized silica gel achieved higher immobilization yield but exhibited lower recovered activity values compared to the silica gel without glutaraldehyde. The enzyme immobilized on both supports showed higher activity at initial substrate concentrations between 400 g L^–1^ and 600 g L^–1^ with different kinetic behaviors. The functionalized silica gel showed greater capacity for reuse over eight consecutive reaction cycles, also exhibiting higher thermal, storage and pH stability compared to the biocatalyst adsorbed onto the pure support. The results suggest a high potential for the application of glutaraldehyde-functionalized silica gel as a support for FTase immobilization for FOS production.

## Graphical abstract

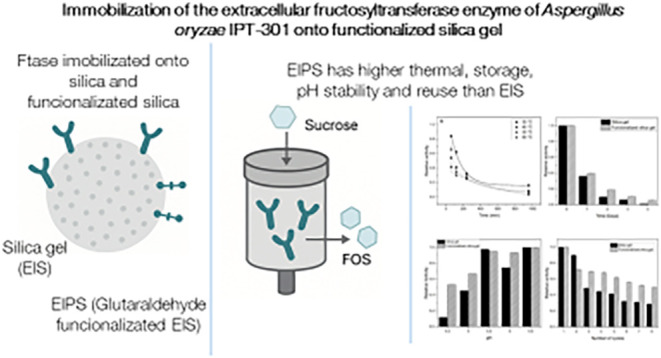

## Introduction

The high added value of fructooligossacharides (FOS) and their global market, estimated at USD 7.2 billion, with a prospect to reach USD 22.92 billion by 2032, make the industrial production of these sugars highly attractive.^1^ However, large-scale production is still limited by the short stability of the soluble fructosyltransferase enzymes used for their synthesis. The costs associated with enzyme production and reaction yield remain challenging in the context of process scaling. To ensure economic feasibility, enzyme recovery and reuse are required.^2^

In this context, enzymatic processes are widely used in the production of oligosaccharides, which can be obtained by the transfructosylation reaction of sucrose molecules catalyzed by microbial enzymes, such as fructosyltransferases (FTase, E.C.2.4.1.9), known for their high transfructosylation activity.^1–3^ FTases are mainly synthesized by fungi of the genera *Aureobasidium*, *Penicillium* and *Aspergillus*.^4,5^ Specifically, *Aspergillus oryzae* IPT-301 has been reported as a promising source of extracellular FTase with high transfructosylation activity among the 17 filamentous fungal strains evaluated.^5^

The production of FOS using soluble FTase can result in high costs for enzyme synthesis and purification, instability of its three-dimensional structure, and possible loss of activity due to process conditions or inhibition by substrate or product.^1^ Studies on the immobilization of extracellular FTase in organic and inorganic supports have been reported in the literature. Yun et al.^6^ immobilized an FTase from *Aureobasidium pullulans* KFCC 10542 onto a highly porous resin, Diaion HPA 25. The immobilized enzyme exhibited greater resistance to pH variations compared to the soluble form and maintained high enzymatic activity for 30 days at 50 °C. Moreover, the optimal temperature (55 °C) and pH (5.5) for FOS production were not affected by the immobilization process. Ghazi et al.^7^ observed high operational activity of commercial enzymes with transfructosylation activity, namely Pectinex Ultra SP-L and Rapidase TF, after immobilization on polymethacrylate-based supports. Platková et al.^8^ evaluated the immobilization of FTases from *A. pullulans* on various commercial supports. The highest specific activities were achieved with Dowex Marathon MSA (Porous Macro Styrene-DVB) and Amberlite IRA 900 (Styrene-DVB), which reached values of 1268 U g^–1^ and 1169 U g^–1^, respectively. Martínez et al.^1^ immobilized an FTase extracted from *Schedonorus arundinaceus* onto Sepabeads and ReliZyme supports by covalent bonds or hydrophobic interactions. The immobilized enzyme showed improved stability under varying pH and temperature conditions, along with higher productivity compared to its soluble form.

Among the numerous materials reported for enzyme immobilization by adsorption, silica gel has been identified as a promising support, since it is abundant in nature, of low-cost, and it exhibits all characteristics of an inorganic support. It stands out for its high mechanical strength, thermal and chemical stability and, mainly, its high specific surface area.^9^ Furthermore, functionalization of the silica gel surface can enhance the resistance of the heterogeneous biocatalyst to denaturing agents (heat, organic solvents and extreme pH), besides minimizing enzyme desorption from the support and enabling its use in different reactor configurations.^10,11^ Previous studies have reported FTase immobilization;^2,12–14^ nonetheless, no studies have been found reporting the immobilization of this enzyme using functionalized silica gel.

One of the most commonly used agents for silica functionalization is glutaraldehyde. The interaction between glutaraldehyde and silica surfaces has been investigated by Paula et al.^15^ and Mendes et al.,^16^ and further elucidated by Barbosa et al.^17^ According to these studies, the maximum enzyme activation is achieved when two glutaraldehyde molecules are present per amino group on the support’s surface. This leads to the formation of a reactive structure capable of interacting with the enzyme’s amino acid residues. Glutaraldehyde acts as a spacer arm by participating in the formation of a covalent bond between the support and the enzyme. It reacts with the amino groups on the support’s surface, generating aldehyde groups that can bind to the enzyme, resulting in stable and active biocatalysts. This method combines high efficiency with a relatively simple activation procedure,^10^ offering advantages in both performance and ease of application.^16,17^

In this context, the present study investigated the immobilization of the extracellular FTase enzyme from *A. oryzae* IPT-301 onto silica gel and glutaraldehyde-functionalized silica gel. The aim was to develop biocatalysts with high enzymatic activity, selectivity, and specificity, along with enhanced thermal and operational stability. These characteristics would enable their application in various reactor configurations for FOS production.

## Materials and Methods

### Treatment and Functionalization of the Immobilization Supports

The silica gel particles (Sigma-Aldrich, 63–200 μm mesh) were washed with 95% P.A ethanol (v v^–1^) at a ratio of 1:5 (support mass: solvent volume) under gentle magnetic stirring for 6 h at room temperature. A fraction of the washed silica gel particles was functionalized with a 25% glutaraldehyde solution (v v^–1^) and 0.2 mol L^–1^ tris-acetate buffer, pH 5.5. Support activation was performed at a ratio of 1:28 (support mass: volume of solution), under gentle magnetic stirring for 18 h at 25 °C. Pure and functionalized silica particles were vacuum-filtered, dried in a kiln at 60 °C for 24 h, and subsequently used in assays of enzyme immobilization by physical adsorption and covalent binding, respectively.

### Production and Immobilization of the Extracellular Microbial Enzyme

The production of the extracellular FTase from *A. oryzae* IPT-310 was conducted according to the procedures described by Cunha et al.^3^ After 64 h of cultivation at 30 °C and 200 rpm,^2,3,18,19^ the culture medium was vacuum-filtered, and 10 mL of permeate (filtered broth) containing an FTase activity of 8 U mL^–1^ were added to an Erlenmeyer flask containing 1 g of the pure silica gel support or the functionalized silica gel support. The immobilization assays were conducted at 175 rpm and 35 °C for 6 h, and the immobilization parameters—yield (IY) and recovered activity (RA)—were calculated according to Faria et al.,^2^ Araújo et al.^13^ and Pereira et al.^20^

### Characterization of the Support and the Heterogeneous Biocatalyst

The morphology of the silica gel particles was examined by Scanning Electron Microscopy (SEM) using an electron microscope (Zeiss EVO MA-10, Germany), at an acceleration voltage of 20 kV and a working distance of 10 mm. The particle size distribution was determined using the software ImageJ. The samples were pretreated in a Vap Prep 61 Sample Degas System and dried for 2 h at 60 °C. Their specific surface areas were measured by nitrogen physisorption at 77 K using a Micromeritics Gemini VII analyzer, according to the Brunauer–Emmett–Teller (BET) method.^21^ Pore size and volume were obtained using the Barrett–Joyner–Halenda (BJH) method.^22^ The chemical bonds and structural characteristics of the heterogeneous biocatalyst, both in the presence and absence of the immobilized enzyme, were analyzed by Fourier transform infrared spectroscopy (FTIR) with an Agilent Cary 630 Spectrometer, operated in the range of 650 to 4000 cm^–1^.

### Enzymatic Activity Assays and Carbohydrate Analysis

The transfructosylation activities (*A*_T_) were measured following the methodology described by Gonçalves et al.,^18^ Faria et al.,^2^ and Garcia et al.^23^ In this procedure, 1.0 g of the immobilized enzyme derivative or 0.1 mL of the soluble enzyme, obtained from the culture broth, was added to a reaction mixture containing 3.7 mL of a 63.6% (m v^–1^) sucrose P.A. solution and 1.2 mL of 0.2 mol L^–1^ tris-acetate buffer at pH 5.5. The reaction was performed in a Dubnoff shaker at 50 °C and 190 rpm for 1 h. It was then stopped by placing the reaction medium in boiling water, followed by rapid cooling on ice to ensure complete enzyme inactivation. One unit (1 U) of transfructosylation activity was defined as the amount of enzyme that transfers 1 μmol of fructose per minute under the specified experimental conditions.^2,3,23^ The concentrations of fructose and transfructosylated fructose in the reaction medium were calculated using a mass balance based on the measured concentrations of glucose and reducing sugars, determined using the enzymatic Glucose kit (GOD-PAP) and the 3,5-dinitrosalicylic acid (DNS) method, respectively.^11,13,20^

### Thermal Stability Assays and Determination of Thermodynamic Parameters

The enzymes immobilized on both supports were evaluated for thermal stability following the methodology described by Faria et al.^2^ and Araújo et al.^13^ The tests were conducted at different temperatures (30 °C, 40 °C, 50 °C, and 60 °C), with all experiments performed in triplicate. The first-order thermal denaturation constant (*k*_D_, in min^–1^) was determined by fitting the model proposed by Sadana and Henley^24^ to the experimental data of residual enzymatic activity as a function of incubation time.^19^ The activation energy of denaturation (*E*_D_, in kJ mol^–1^) of the enzyme was determined using the Arrhenius equation.^3,12,23^ Half-life (*t*_1/2_, in min), enthalpy of activation of denaturation (Δ*H*_D_, in kJ mol^–1^), Gibbs energy of activation of denaturation (Δ*G*_D_, in kJ mol^–1^), and entropy of activation of denaturation (Δ*S*_D_, in kJ mol^–1^ K^–1^) were determined employing eqs 1–4, respectively, where *h* is the Planck’s constant (11.04 × 10–36 J min^–1^) and *k*_b_ is the Boltzmann’s constant (1.38 × 10–23 J K^–1^).^3,13,23^

1 $$t_{1/2}=-\frac{1}{\mathrm{kd}}\ln\left(\frac{\frac{1}{2}-\alpha }{1-\alpha }\right)$$

2 $$\Delta H_{D}=E_{D}-\mathrm{RT}$$

3 $$\Delta G_{D}=-\mathrm{RT}\ln\left(\frac{k_{D}h}{k_{b}T}\right)$$

4 $$\Delta S_{D}=\frac{\Delta H_{D}-\Delta G_{D}}{T}$$

### Effect of the Substrate on Enzymatic Activity and Determination of the Kinetic Parameters

The transfructosylation activity of the heterogeneous biocatalyst (enzyme immobilized on silica gel and functionalized silica gel) was determined at 50 °C using 3.7 mL of a sucrose P.A. solution at different concentrations (200, 300, 400, 470, 500, and 600 g L^–1^) plus 1.2 mL of tris-acetate buffer at 0.2 mol L^–1^ and pH 5.5. The kinetic parameters (maximum reaction rate—*V*_max_, apparent dissociation constant—*K*_0.5_, and Hill coefficient—*n*) were obtained by nonlinear regression analysis fitting the Hill model to the experimental data.

### Operational Stability Assays

The operational stability assays were performed according to Faria,^2^ Araújo^13^ and Pereira.^20^ Approximately 1 g of heterogeneous biocatalyst (enzyme immobilized on silica gel and functionalized silica gel) was added to the reaction medium containing 3.7 mL of sucrose P.A. at 47% (w/v) and 1.2 mL of tris-acetate buffer at 0.2 mol L^–1^ (pH 5.5). The enzymatic reaction was performed under standard conditions. At the end of each batch cycle (1 h of reaction), the heterogeneous biocatalyst was removed from the reaction medium by vacuum filtration and reintroduced into a fresh reaction medium of similar composition. The transfructosylation activities were evaluated as a function of the number of enzymatic reaction cycles.

### pH and Storage Stability Assays

The stability at different pH values was determined by the incubation of the heterogeneous biocatalyst (enzyme immobilized on silica gel and functionalized silica gel) in tris-acetate buffers at 0.2 mol L^–1^, at a pH interval of 4.5–6.5 in the absence of substrate, at 4 °C, for 24 h, and measuring the residual activity under standard conditions. The storage stability assays were performed with the heterogeneous biocatalyst for 4 days. The immobilized FTase was maintained in 0.2 mol L^–1^ tris-acetate buffer (pH 5.5), in the absence of substrate, under refrigeration at 4 °C, and the enzymatic activities were monitored daily under standard conditions.

### Statistical Analysis

All experiments were performed in triplicate. The mean comparisons were calculated using the Tukey’s Honest Significant Difference (HSD) test at a 95% confidence interval.

## Results and Discussion

### Characterization of the Suport and the Heterogeneous Biocatalyst

Scanning electron microscopy (SEM) images of the pure silica gel particles before enzyme immobilization are shown in Figure 1 at different magnifications. The particles have a size range from 102 to 256 μm (Figure 1a). It was also observed that the particles are uniform (Figure 1b), and at higher magnifications (Figure 1c,d), low sphericity and subangular rounding were observed. This is a positive aspect for enzyme immobilization, because higher surface roughness of the support material favors adsorption due to the increase in the specific surface area available for enzyme binding and nonspecific interactions.^25^

**Figure 1:**
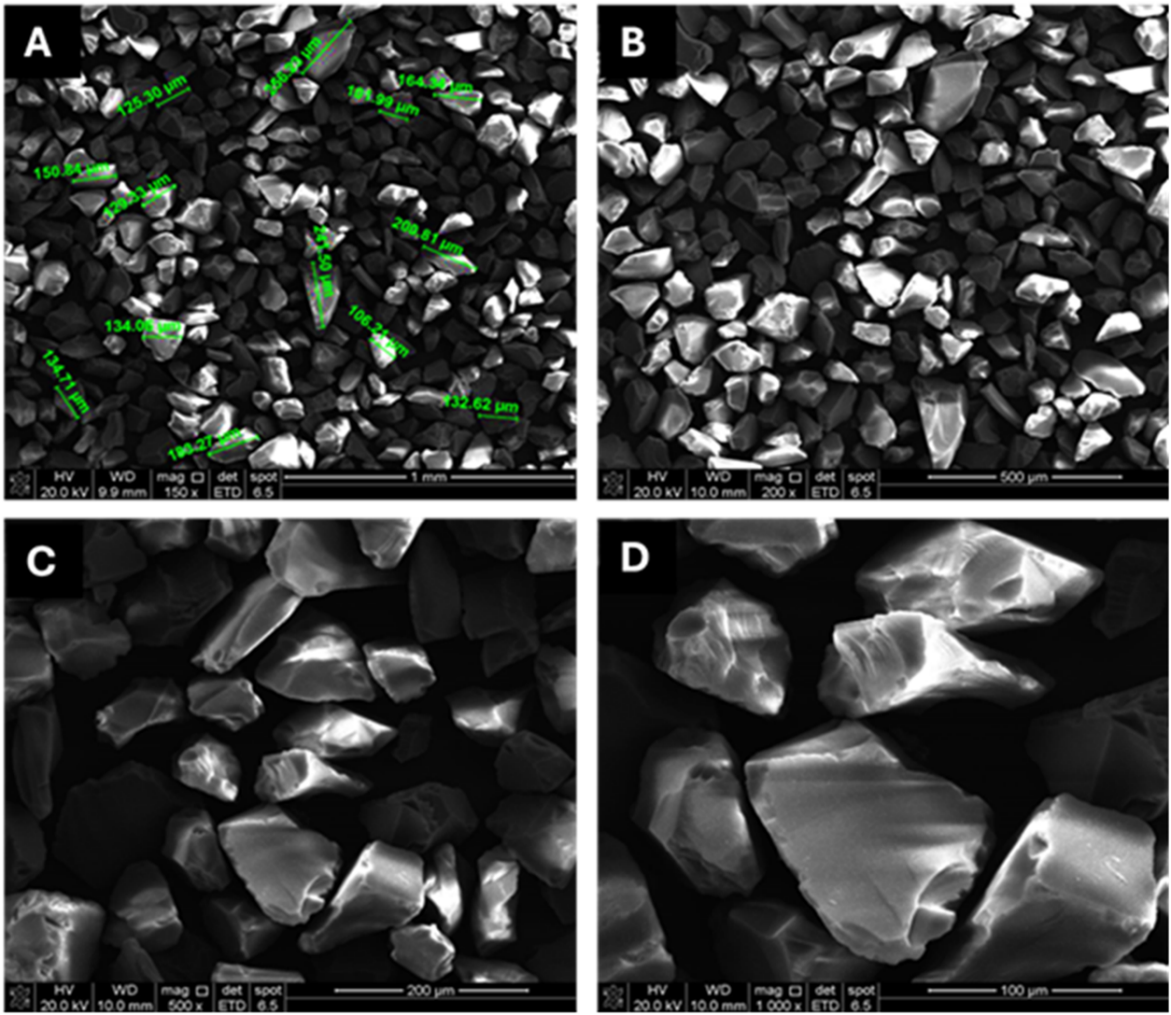
Electronic micrographs of enlarged silica gel particles samples at different magnifications: (A) 150×; (B) 200×; (C) 500× and (D) 1000×.

Table 1 shows the results obtained for specific surface areas, as well as pore size and volume, for the silica gel and functionalized silica gel particles. Using the BET technique, a reduction in the specific surface area of the support by approximately 1.6 times was observed when silica gel particles were subjected to functionalization. The introduction of organic groups, specifically glutaraldehyde molecules, onto the silica surface via reactions with silanol groups leads to the formation of structures that can partially coat both the external surface and the internal pore walls of the material. This coating affects the adsorption behavior evaluated by the BET technique, resulting in a reduction of the specific surface area,^13,17,18^ despite minimal changes in total porosity, as indicated by the pore size values (Table 1), which appear partially coated or blocked. Moreover, an approximate 52% decrease in pore volume was observed following support functionalization, further supporting the hypothesis of partial pore blockage or filling by the functional groups derived from glutaraldehyde molecules. Following the BJH method,^26^ similar pore sizes were obtained for pure (56.92 Å) and functionalized (59.04 Å) silica gel particles, both classified as mesoporous (pore sizes between 20 and 500 Å).^27^ Mesoporous silica gel, due to its large specific surface area, high porosity, and large pore volume, provides versatility in adsorbing specific compounds.^28^ The results of the physical characterization showed that the functionalization process did not significantly affect the textural properties of the support, corroborating its characteristic of mechanical strength and, above all, its thermal and chemical stabilities.^9^

**Table 1:** Characterization of the Pure and Functionalized Silica Gel Particles Employing the BET and BJH Methods

support	specific surface area (m^2^ g^–1^)	pore size (Å)	pore volume (cm^3^ g^–1^)
silica gel	319.69	56.92	7.50 × 10^–2^
functionalized silica gel	198.93	59.04	3.59 × 10^–2^

The FTIR spectra of the silica gel support (sample A), the FTase immobilized onto silica gel (sample B), and the FTase immobilized onto the glutaraldehyde-functionalized silica gel (sample C) are shown in Figure 2.

**Figure 2:**
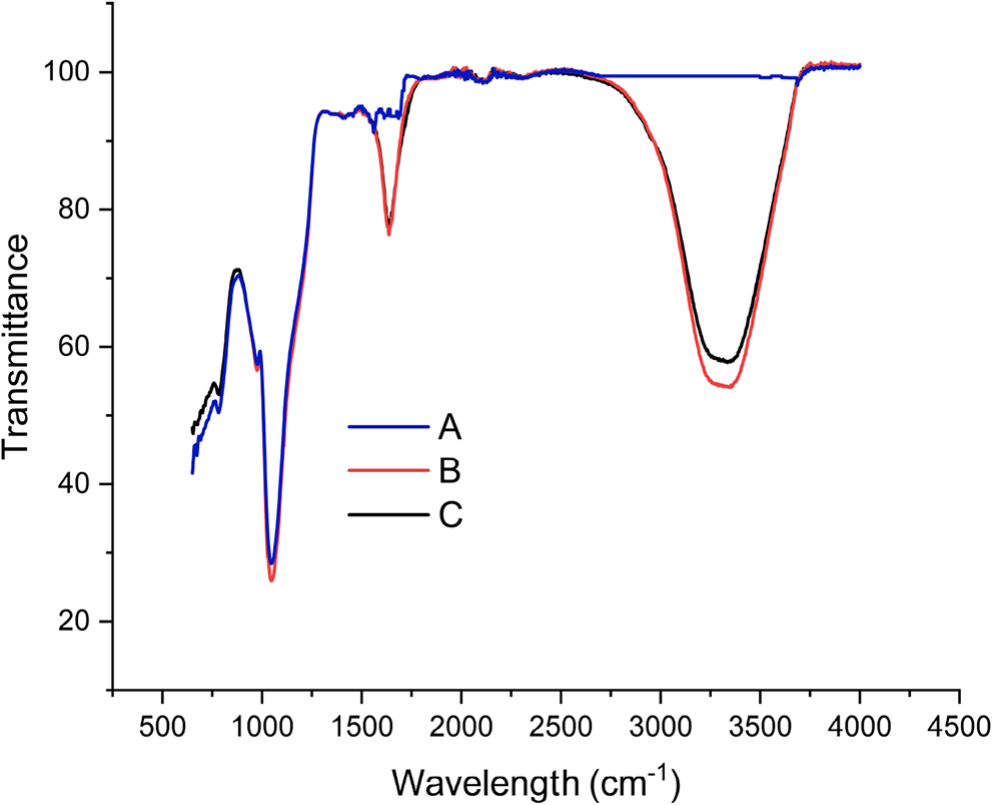
FTIR spectra of samples A (silica gel), B (immobilized enzyme onto silica gel) and C (immobilized enzyme onto functionalized silica gel).

The infrared spectrum (Samples A, B, and C) exhibited bands in the regions of 1050 cm^–1^, 970 cm^–1^ and 800 cm^–1^, respectively. The peaks of 1050 and 800 cm^–1^ correspond to the asymmetric and symmetric vibrations of Si–O–Si, respectively.^29–31^

The functionalized silica gel supports containing immobilized FTase presented two active regions in the infrared spectrum, at 3300 cm^–1^ and 1630 cm^–1^. The band in the region 3300 cm^–1^ corresponds to the stretching vibrations of the N–H groups (amines) in the enzyme, while the band around the region 1630 cm^–1^ corresponds to amide vibrations (C–O stretch, C–N stretch, and N–H bending). The band at the region 3300 cm^–1^ extends from 3700 cm^–1^ to about 3000 cm^–1^, whose normalized intensity is higher than that of the band at 1630 cm^–1^. According to Socrates,^32^ the amine band should exhibit higher intensity, which is not observed in Figure 2. The enhanced intensity of the N–H stretching band (amines) may derive from overlapping bands, including O–H stretching around 3270 cm^–1^. Although the samples were carefully dried before FTIR analysis, a fraction of water may have remained adsorbed on the silica gel surface due to the presence of Si–OH groups. These groups have strong affinity for water molecules, which can explain the peak around 3300 cm^–1^, assigned to O–H stretching overlapping with the N–H vibration of the enzyme.^33–35^ Such behavior is commonly reported for porous materials, including functionalized silica gel.^32^ In contrast, the absence of these bands around 3300 cm^–1^ on the immobilized surface may be attributed to glutaraldehyde-mediated immobilization, which restricts the mobility of the enzyme’s amine (N–H) groups, reducing the intensity of vibrational bands and making them less evident in FTIR analysis. Furthermore, the lower retention of water molecules on the functionalized surface, combined with possible overlap with other bands of the support or the linking agent, may also contribute to the attenuation or absence of the broad band in this region.^36^

The results confirm the presence of organic groups of the enzyme on the surface of the silica gel and the functionalized silica gel after biocatalyst immobilization. Silica is presented in tetrahedral units of SiO_4_^–2^, randomly distributed and joined by siloxane bridges, Si–O–Si, inside it, and contains vicinal silanol groups, Si–OH, and geminals, HO–Si–OH, dispersed on the surface, which are sensitive to the reactions that make possible the chemical modifications of this matrix. Glutaraldehyde builds bonds with these siloxane groups, and binds with the amino groups of the enzyme, adding a covalent bond between the enzyme and the support.^37^

### Enzyme Immobilization Parameters

The immobilization parameters were determined for the extracellular FTase immobilized onto silica gel and onto the glutaraldehyde-functionalized silica gel (Table 2).

**Table 2:** Immobilization Parameters of the Extracellular FTase Adsorbed on the Silica Gel and Functionalized Silica Gel Supports^a^

	immobilization parameters (%)	
support	immobilization yield (IY)	recovered activity (RA)
silica gel	12.33 ± 1.15	9.67 ± 0.57
functionalized silica gel	39.25 ± 1.72	6.76 ± 0.65

a Immobilization assays conducted at 35 °C, pH 5.5 and 175 rpm, with a ratio of 1:10 (support mass: culture broth volume).

The results showed an expressive increase in immobilization yield (IY), around 3.5 times higher when the support was functionalized, indicating that the insertion of reactive groups and/or aldehyde spacer arm on the surface of the silica gel particles favored enzyme immobilization by covalent binding.^10^ Paula et al.^15^ indicated that the most effective support activation occurs when two glutaraldehyde molecules are available per amino group on the support surface. This configuration leads to the formation of a reactive structure with the enzyme’s amino acid residues, which helps protect the protein from environmental factors and facilitate stronger binding between the biomolecule and the support. The glutaraldehyde molecule acts as a bridge, reacting with both the support and the enzyme. As a result, the enzyme becomes covalently bound to the support by interactions between its amino groups and the aldehyde groups of the functionalized surface, forming Schiff base linkages.^10,17^ Although the functionalization of the support increased immobilization yield, this parameter may have been influenced by impurities present in the culture broth, such as amino acids, low molecular weight polypeptides, traces of mineral and carbohydrates. These substances can adsorb onto the support’s surface or interact with the enzyme, thereby affecting its immobilization.^13,20,23^

On the other hand, the FTase immobilized onto silica gel exhibited a recovered activity (RA) 1.5 times higher than that observed for the FTase immobilized onto the functionalized silica gel (Table 2). This suggests the functionalization of the inorganic support with glutaraldehyde caused a partial reduction in enzymatic activity, which can be attributed to conformational changes in the enzyme, modifications in its active site, or increased rigidity caused by the covalent bonds with reactive groups present in the silica gel.^13,38^ Covalent immobilization can result in stronger binding between the biocatalyst and the support, increasing molecular rigidity and potentially reducing catalytic activity.^10^ In addition, the enzyme’s RA might have been affected by steric effects caused by the functionalization of the silica gel surface, which may induce conformational changes in the three-dimensional structure of the FTase. Furthermore, in hydrophilic supports such as silica gel, the formation of aggregates and three-dimensional structures among the particles may cause diffusional restrictions of the substrate to the active site of the immobilized enzyme.^39,40^

Vescovi et al.^39^ investigated the immobilization of lipases from *Thermomyces lanuginosus* (TLL) and *Pseudomonas fluorescens* (PFL) on chemically modified silica particles. The silica surfaces were functionalized with octyl (OS), octyl–glutaraldehyde (OSGlu), octyl–glyoxyl (OSGlx), and octyl–epoxy (OSEpx) groups. The immobilization yield of TLL was higher on the functionalized supports, indicating the formation of a dense enzyme layer on all modified silica particles. Araújo et al.^13^ reported the immobilization of an FTase from *A. oryzae* IPT-301 on polyhydroxybutyrate (PHB) and glutaraldehyde-activated polyhydroxybutyrate (GLU-PHB), achieving immobilization yields of 41% and 55%, respectively. The recovered activity of the FTase adsorbed on PHB (17%) was higher compared to that of the biocatalyst immobilized on GLU-PHB (11%). In contrast, previous studies by Garcia et al.^41^ achieved immobilization yield and recovered activity of 57.7% and 87.8%, respectively, for the FTase from the same microorganism immobilized on silica-niobia support.

### Effect of Sucrose Concentration on Enzyme Activity and Kinetic Parameters

The influence of sucrose concentration on the transfructosylation activity of the extracellular FTase immobilized onto silica gel and glutaraldehyde-functionalized silica gel is shown in Figure 3.

**Figure 3:**
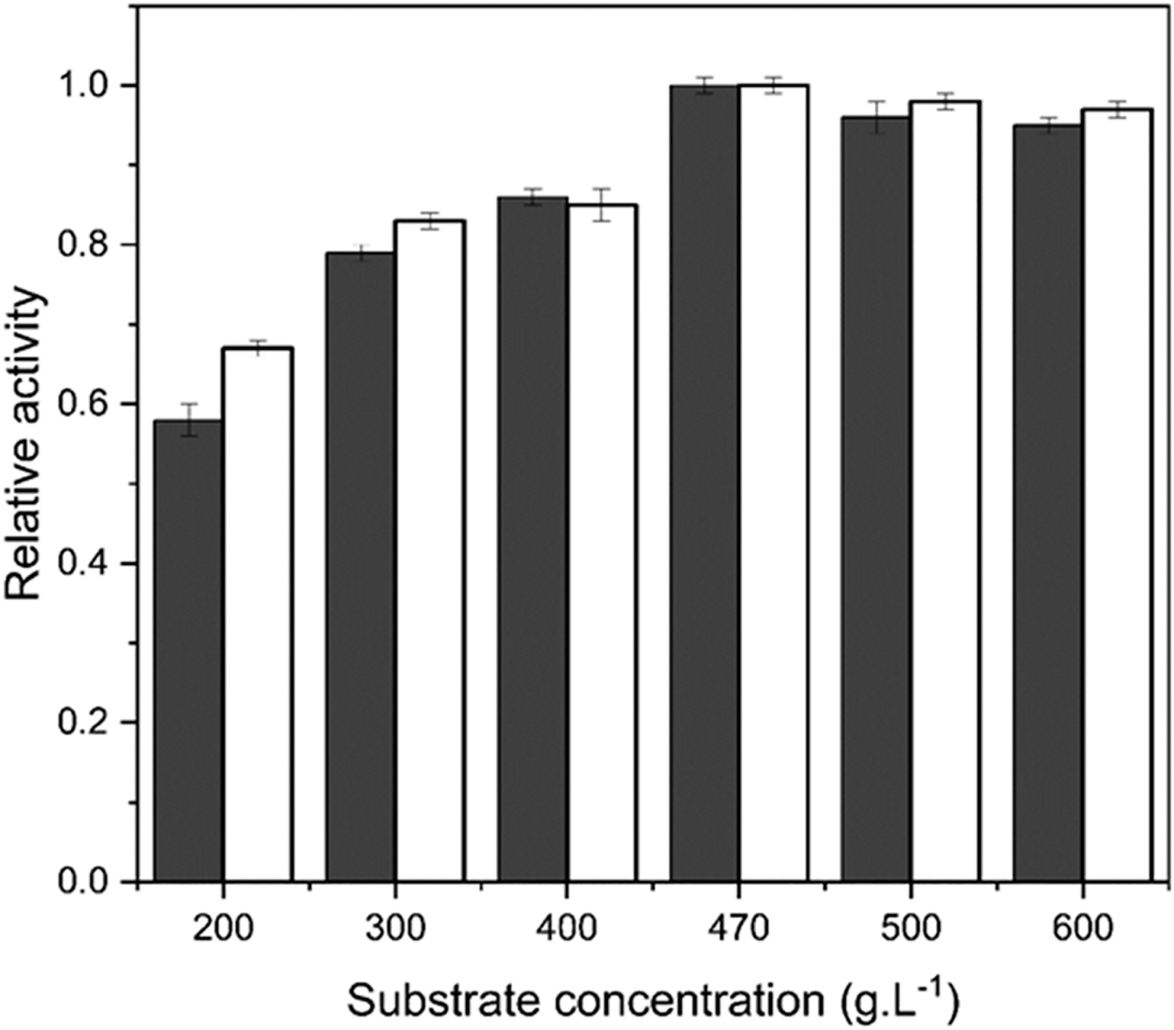
Effect of substrate concentration on the transfructosylation activity of extracellular Ftase from *A*. *oryzae* IPT-301 immobilized onto silica gel (unfilled bar) and glutaraldehyde-functionalized silica gel (filled bar). Reaction conditions: 20, 30, 40, 47, 50, and 60 (w v^–1^) sucrose solution and 0.2 mol L^–1^ of tris-acetate buffer (pH 5.5), 190 rpm at 50 °C for 60 min. The maximum activity for FTase immobilized onto silica gel (4.52 ± 0.11 U g^–1^) and FTase immobilized onto glutaraldehyde-functionalized silica gel (4.56 ± 0.15 U g^–1^) was defined as 100% of relative activity.

The highest activity (*A*_T_) values were obtained at sucrose concentrations above 400 g L^–1^, with the maximum activity (4.5629 ± 0.0015) achieved at 470 g L^–1^ for both supports. Similarly, Faria et al.^2^ reported that the FTase from *A. oryzae* IPT-301 immobilized onto silica gel also showed the highest *A*_T_ at 470 g L^–1^. In turn, a previous study performed by Araújo et al.^13^ with the same enzyme immobilized on polyhydroxybutyrate and glutaraldehyde-activated polyhydroxybutyrate showed *A*_T_ at substrate concentrations between 400 and 500 g L^–1^. The results suggest that the influence of sucrose concentration on the enzymatic activity was not altered by its immobilization on organic and inorganic supports functionalized with glutaraldehyde.

At substrate concentrations above 500 g L^–1^, a slight decrease in enzymatic activity was observed, likely due to the inhibition of the biocatalyst’s active sites by sucrose. Similarly, Cunha et al.^3^ reported a decline in the transfrtuctosylation activity of an extracelullar FTase from *A. oryzae* IPT-301 under comparable condtions. In turn, Alvarado-Huallanco and Maugeri-Filho^42^ found that high sucrose concentrations (up to 70% w v^–1^) caused substrate inhibition in the soluble FTase from *Rhodotorula sp*. Finally, the lowest A_T_ for both immobilized FTases were observed at 200 g L^–1^ of sucrose (Figure 3), which can be attributed to a limited availability of substrate molecules, affecting the progression of the catalytic reaction.^2,13,20^

Figure 4 shows the fit of the Hill model to the A_T_ data of the FTase immobilized onto silica gel and functionalized silica gel at different substrate concentrations. The high regression coefficient values showed that the Hill model (*R* ^2^ = 0.994 for the FTase immobilized onto functionalized silica gel and *R* ^2^ = 0.90 for the FTase immobilized onto silica gel) demonstrate that the Hill model best describes the variations in enzymatic activity with substrate concentration. According to Ghazi et al.,^7^ enzymes with fructosyl group transfer activity are generally well described by Hill’s kinetics, indicating the presence of cooperativity phenomena between enzyme and substrate.

**Figure 4:**
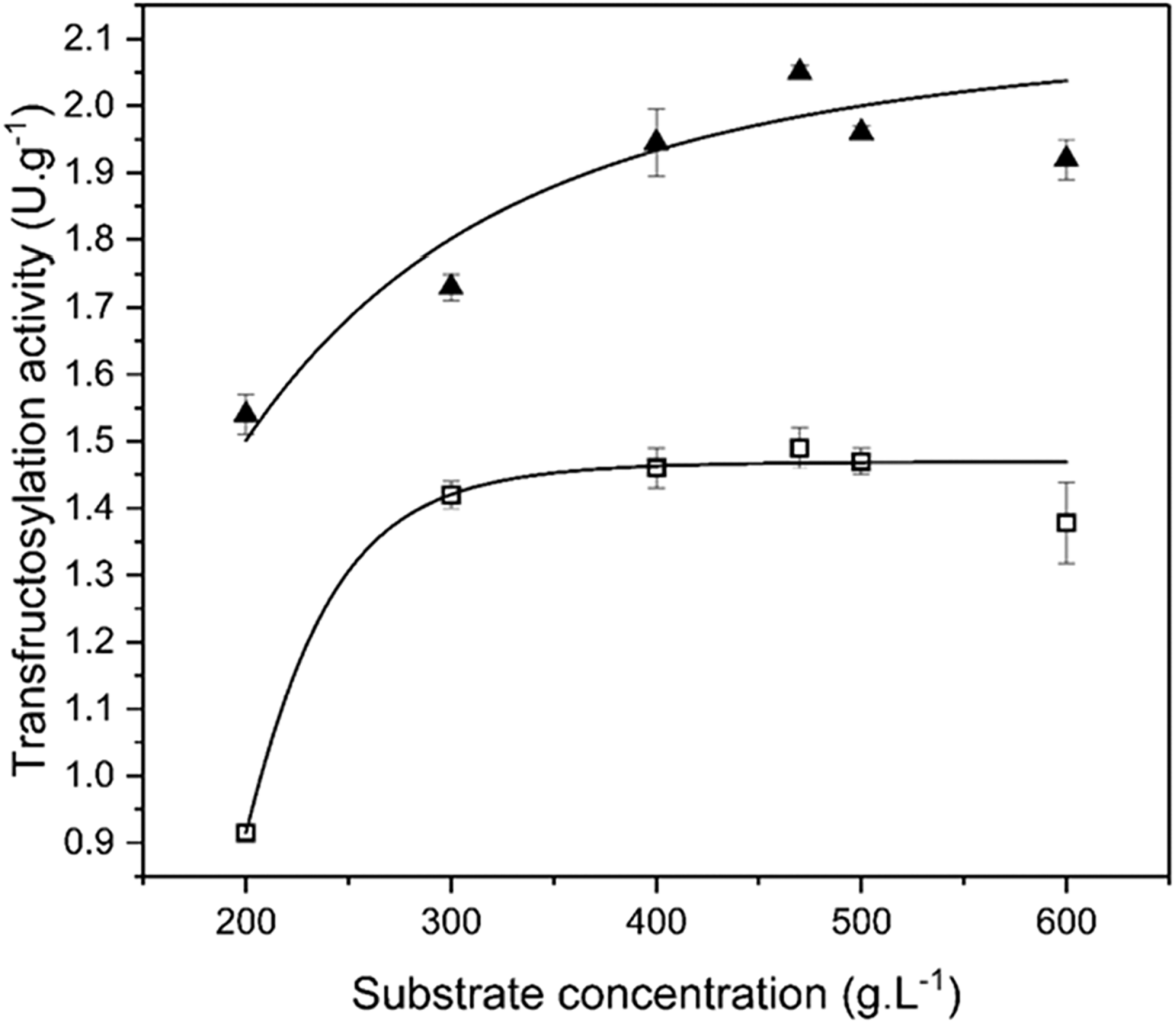
Data on the transfructosylation activity and its fit to the Hill model (continuous line) for the determination of the kinetic parameters of the FTase from *A. oryzae* IPT-301 immobilized onto silica gel (triangle) and functionalized silica gel (square).

The *V*_max_ values obtained were 2.56 U g^–1^ and 2.52 U g^–1^ for the FTase immobilized onto silica gel and functionalized silica gel, respectively. These low values for both cases can be attributed to limited mass transport to the support into the support pores, which hinders the substrate from reaching the enzyme’s active site.^3,13^ In turn, *K*_0.5_ values of 186 g L^–1^ and 129 g L^–1^ were obtained for the biocatalyst immobilized on silica gel and functionalized silica gel, respectively. This kinetic parameter represents the sucrose concentration at which the reaction rate reaches half of its maximum value; lower *K*_0.5_ values indicate greater substrate affinity for the enzyme’s active sites.^42^ These results suggest that the covalent attachment of the enzyme to the support led to a reduction in the affinity between FTase and sucrose molecules. Similarly, a *K*_0.5_ value was reported for the FTase from *A. oryzae* IPT-301 adsorbed on silica gel (204.2 g L^–1^),^2^ polyhydroxybutyrate (221.14 g L^–1^), and glutaraldehyde-activated polyhydroxybutyrate (205.9 g L^–1^).^13^ Finally, the Hill coefficients (*n*) obtained (values greater than 1 in both cases) indicated positive cooperativity between the substrate and the enzyme’s active sites. This is characterized by an increased affinity following the binding of the first molecule to the active site, a phenomenon commonly observed in enzymes with multiple active sites and subunits.^42^

### Thermal Stability of the Enzyme Immobilized in Silica Gel and in the Functionalized Silica Gel

The thermal stability of the FTase immobilized onto silica gel and funcionalized silica gel was evaluated at 30, 40, 50, and 60 °C for a maximum incubation period of 16 h (Figure 5). The first order thermal deactivation constants (*k*_D_) at each temperature were obtained by nonlinear regression, using the Sadana and Henley model,^24^ while the activation energy of denaturation (*E*_D_) was determined from the linearized Arrhenius Equation.^13,18–20^

**Figure 5:**
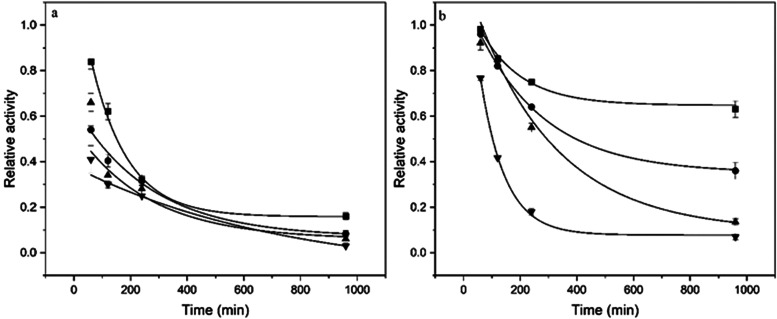
Thermal stabilities of FTase immobilized onto functionalized silica gel (a), and FTase immobilized onto silica gel (b) over 16 h of incubation at different temperatures: 30 °C (■), 40 °C (●), 50 °C (▲), and 60 °C (▼). Continuous line: Sadana and Henley thermal inactivation model fitted to the experimental data. Reaction conditions: 47% (w v^–1^) sucrose solution and 0.2 mol L^–1^ of tris-acetate buffer (pH 5.5), 190 rpm at 50 °C for 60 min. The maximum activity for FTase immobilized onto functionalized silica gel (4.33 ± 0.06 U g^–1^), and FTase immobilized onto silica gel (6.17 ± 0.16 U g^–1^) were defined as 100% of relative activity.

The FTase immobilized onto the functionalized silica gel showed greater retention of the transfructosylation activity compared to the biocatalyst adsorbed onto silica gel, with the greatest retention observed at 30 °C. At this temperature, the FTase immobilized onto the functionalized silica gel retained over 70% of this activity after 16 h of incubation, whereas the enzyme immobilized onto silica gel retained only 20% under the same conditions. Additionally, after 4 h of incubation at 40 and 50 °C, the FTase immobilized onto the functionalized silica gel retained more than 50% of its activity, while the enzyme immobilized on the modified support retained only 30%. At 60 °C, the greatest reductions in enzymatic activity were observed across all incubation periods, indicating that the FTase immobilized by both physical adsorption and covalent binding underwent thermal denaturation, retaining around 10% of its initial activity after 16 h of incubation. Usually, the exposure of enzymes to high temperatures results in an irreversible loss of their catalytic properties, since there is a cleavage of noncovalent interactions and conformational changes.^43,44^ The results suggest the immobilization of FTase by covalent bonding provided the enzyme with a higher binding strength and, hence, greater resistance to denaturation by heat.^44^

Silva et al.^19^ evaluated the thermal stability of the soluble FTase from *A. oryzae* IPT-301 at 30–60 °C for 8 h, reporting up to 80% of residual activity at 30 °C. Previous thermal stability studies with the same enzyme immobilized on inorganic and organic supports have also been reported. Faria et al.^2^ observed activities decreasing from 90% to 40% after 17 h at similar temperatures when immobilized onto silica gel. Araújo et al.^13^ reported residual activity from 70% to 20% for the FTase immobilized on polyhydroxybutyrate, and from 75 to 30% for the enzyme immobilized on the glutaraldehyde-activated polyhydroxybutyrate support after 24 h of incubation at temperatures between 30 and 60 °C, respectively. In turn, Pereira et al.^20^ reported residual transfructosylation activities of 18%, 15%, and 5% at 40, 50, and 60 °C, respectively, for the enzyme immobilized onto alkali-treated corncob particles.

The thermodynamic parameters of the FTase immobilized onto silica gel and glutaraldehyde-functionalized silica gel are shown in Table 3.

**Table 3:** Thermodynamic Parameters of the FTase Immobilized onto Silica Gel (S) and Glutaraldehyde-Functionalized Silica Gel (GLU-S), Incubated at Different Temperatures^a^,^b^

		temperature (°C)			
parameters	immobilized FTase	30	40	50	60
*R* ^2^ (Sadana and Henley model)	S	0.77	0.82	0.77	0.86
	GLU-S	0.98	0.99	0.98	0.99
*k*_D_ (min^–1^)	S	4.2 × 10^–3^	4.9 × 10^–3^	5.0 × 10^–3^	5.8 × 10^–3^
	GLU-S	0.6 × 10^–3^	1.9 × 10^–3^	2.1 × 10^–3^	9.6 × 10^–3^
*t*_1/2_ (min)	S	164.00	141.44	136.79	118.45
	GLU-S	1089.77	351.51	316.88	71.85
*E*_D_ (kJ mol^–1^)	S	8.47			
	GLU-S	69.15			
Δ*H*_D_ (kJ mol^–1^)	S	5.95	5.86	5.78	5.70
	GLU-S	66.6	66.5	66.46	66.38
Δ*G*_D_ (kJ mol^–1^)	S	98.38	101.33	104.56	107.48
	GLU-S	103.16	103.70	106.81	106.09
Δ*S*_D_ (kJ mol K^–1^)	S	–0.30	–0.30	–0.30	–0.30
	GLU-S	–0.12	–0.11	–0.12	–0.11

a The immobilized FTases were incubated at pH 5.5 (0.2 mol L–1 tris-acetate buffer) in the absence of the substrate, at 30, 40, 50, and 60 °C.

b S: silica gel support; GLU-S: glutaraldehyde-functionalized silica gel; *R* ^2^: correlation coefficient for the *k*_D_ values; *k*_D_: first-order thermal denaturation constant; *t*_1/2_: half-life of the biocatalyst; *E*_D_: activation energy of denaturation; Δ*H*_D_: enthalpy of activation of denaturation; Δ*G*_D_: Gibbs energy of activation of denaturation; Δ*S*_D_: entropy of activation of denaturation.

The half-life (*t*_1/2_) of both biocatalysts decreased progressively, while the first-order thermal denaturation constant (*k*_D_) increased steadily with the rise in incubation temperature. Half-life refers to the time required for an enzyme’s activity to decline to 50% of its initial value at a specified temperature.^45^ According to Martínez et al.,^1^ half-life is a key parameter for assessing thermal stability: the longer the half-life, the greater the enzyme stability. This parameter also has economic relevance, since it influences the feasibility of reusing the biocatalyst in FOS synthesis. The FTase immobilized on the glutaraldehyde-functionalized silica gel exhibited *t*_1/2_ values approximately 6.6-fold and 2.5-fold higher than those of the FTase immobilized onto silica gel at 30 °C and 40–50 °C, respectively, indicating greater thermostability when bound to the functionalized inorganic support. The longer half-life observed for the activated silica gel can be attributed to the covalent bonds formed between the enzyme and glutaraldehyde, which are stronger than the adsorption interactions that occur when the enzyme is bound to unmodified silica gel.^40^

Table 3 also shows the values of the activation energy of denaturation (*E*_D_) for both biocatalysts. The *E*_D_ values indicate that the FTase immobilized onto functionalized silica gel exhibited energy levels 8.3 times higher than those of the enzyme immobilized onto silica gel, suggesting that more energy in the form of heat is required to denature the biocatalyst under the tested conditions. This parameter represents the minimum amount of energy required for an enzyme to become inactive.^3,13,20^ The thermal denaturation of enzymes involves irreversible conformational changes and requires the input of activation energy.^40,45^ Therefore, a higher *E*_D_ value is indicative of greater biocatalyst thermostability. Similarly, higher and positive values of the enthalpy of activation of denaturation (Δ*H*_D_) were observed for the FTase immobilized onto functionalized gel across the entire incubation temperature range investigated, suggesting the biocatalyst exhibited greater thermal stability compared to the enzyme immobilized by physical adsorption on the inorganic support. Within the evaluated temperature range, the FTase immobilized onto functionalized silica gel showed Δ*H*_D_ values 11.5 times higher than those of the biocatalyst adsorbed on silica gel, indicating greater thermal resistance of the FTase due to its covalent bonding with the support.

Table 3 also shows the Gibbs energy of activation of denaturation (Δ*G*_D_) and entropy of activation of denaturation (Δ*S*_D_) values. The Δ*G*_D_ values were positive in all cases, indicating that, at equilibrium, the concentration of the biocatalyst in the native state is higher than in the denatured state. Consequently, for both immobilized biocatalysts, the transition from the native to the denatured states was a nonspontaneous process.^18,40^ Therefore, higher Δ*G*_D_ values correspond to greater thermostability of the immobilized FTase. This thermodynamic parameter is regarded as the most reliable indicator of enzyme stability, since it integrates both enthalpic and entropic contributions and reflects the overall molecular resistance to unfolding.^45^ Finally, the negative Δ*S*_D_ values observed for the immobilized FTase on both supports indicate a transition of the enzyme to a more ordered and stable conformation. This occurs because the enzyme’s resistance to unfolding—driven by strengthened hydrophobic interactions—overcomes its tendency to dissociate due to weakened polar interactions at elevated temperatures.^45–47^ The results indicate that the immobilization of FTases from *A. oryzae* IPT-301 on glutaraldehyde-functionalized silica gel improves thermal stability, making them better suited for biocatalytic processes at elevated temperatures.

### pH Stability and Storage Stability

The pH stability of the FTase immobilized onto silica gel and on the glutaraldehyde-functionalized silica gel is shown in Figure 6.

**Figure 6:**
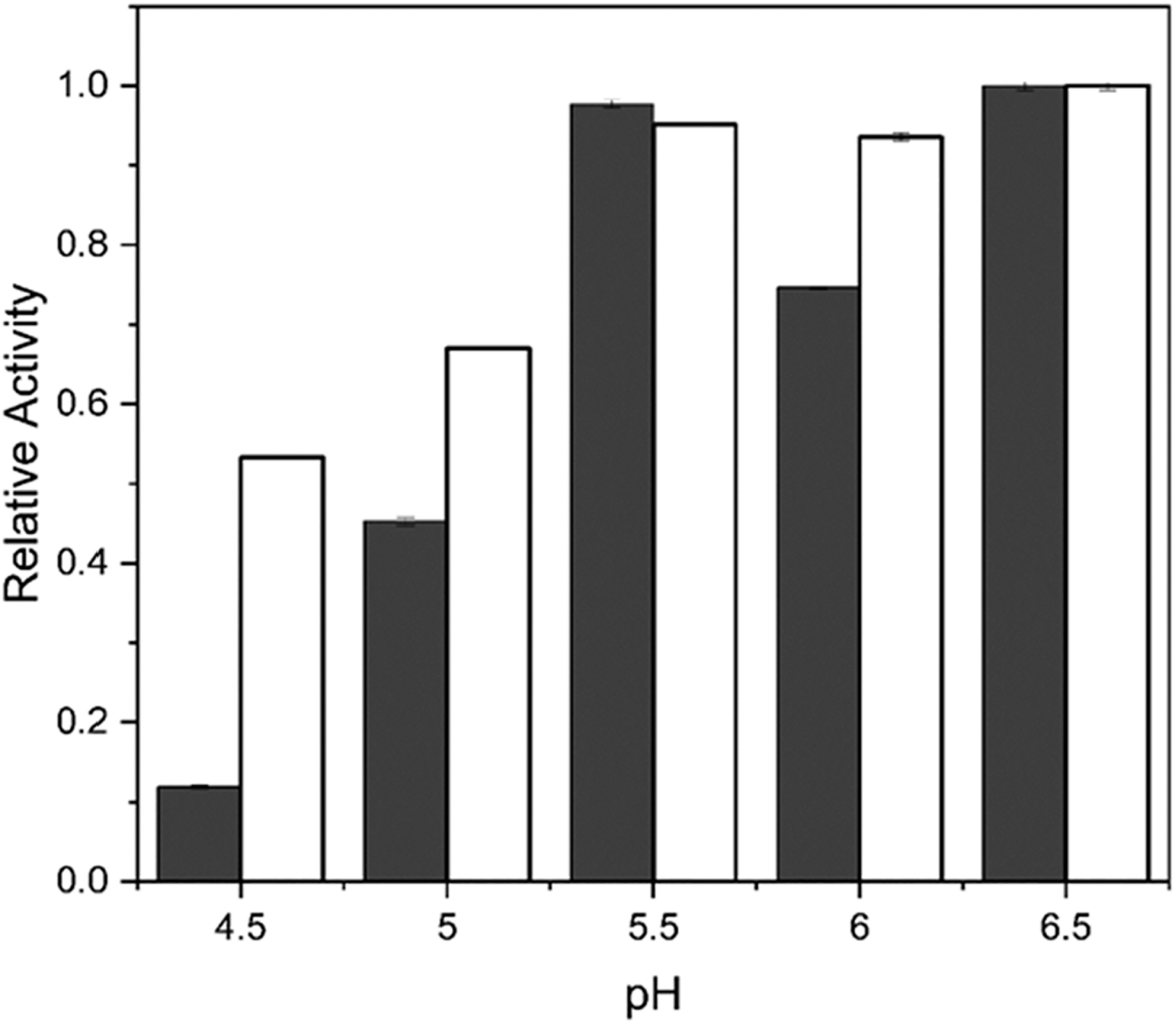
Stability of FTase immobilized onto silica gel (unfilled bar) and onto glutaraldehyde-functionalized silica gel (filled bar) after 24 h of incubation at 4 °C in tris-acetate buffer solutions at different pH values. Reaction conditions: 47% (w v^–1^) sucrose solution and 0.2 mol L^–1^ of tris-acetate buffer (pH 5.5), 190 rpm at 50 °C for 60 min. The maximum activity for FTase immobilized onto functionalized silica gel (4.99 ± 0.02 U g^–1^), and FTase immobilized onto silica gel (7.43 ± 0.03 U g^–1^) were defined as 100% of relative activity.

On both supports, the biocatalyst exhibited high resistance within the pH range of 5.5–6.5, maintaining the relative activity above 70% for the enzyme immobilized onto silica gel, and above 90% for that immobilized on the functionalized support. Enzyme immobilization protects the reactive groups of the protein structure from pH-induced alterations, and immobilized enzymes are generally more robust and resistant to pH fluctuations in the reaction medium.^16^ For the FTase immobilized onto functionalized silica gel, the reaction between glutaraldehyde molecules and the enzyme’s functional groups may induce conformational changes and alter the ionization state of the biocatalyst’s microenvironment, thereby enhancing pH stability.^41,44^

On the other hand, at more acidic pH values (4.5–5.0), the lowest relative activity levels were observed, particularly for the FTase adsorbed on the unmodified silica gel. Enzymatic inactivation by pH involves the unfolding of the protein molecule due to changes in the electrostatic balance and hydrogen bonds, which increase as a result of a change in the ionization state of the enzyme’s ionogenic group caused by pH.^13^ The results indicate that support functionalization with glutaraldehyde improves biocatalyst stability by the formation of covalent bonds.^37^ Additionally, amino groups exhibit high reactivity to the aldehyde groups of glutaraldehyde, contributing to the formation of stable covalent bonds during enzyme immobilization.^48^ Enzymes that are stable over a wide pH range offer the advantages of reducing storage cost and being viable for industrial applications.^49^

The biocatalysts were stored at pH 5.5 for 4 days (Figure 7), and their relative activity was monitored throughout this period.

**Figure 7:**
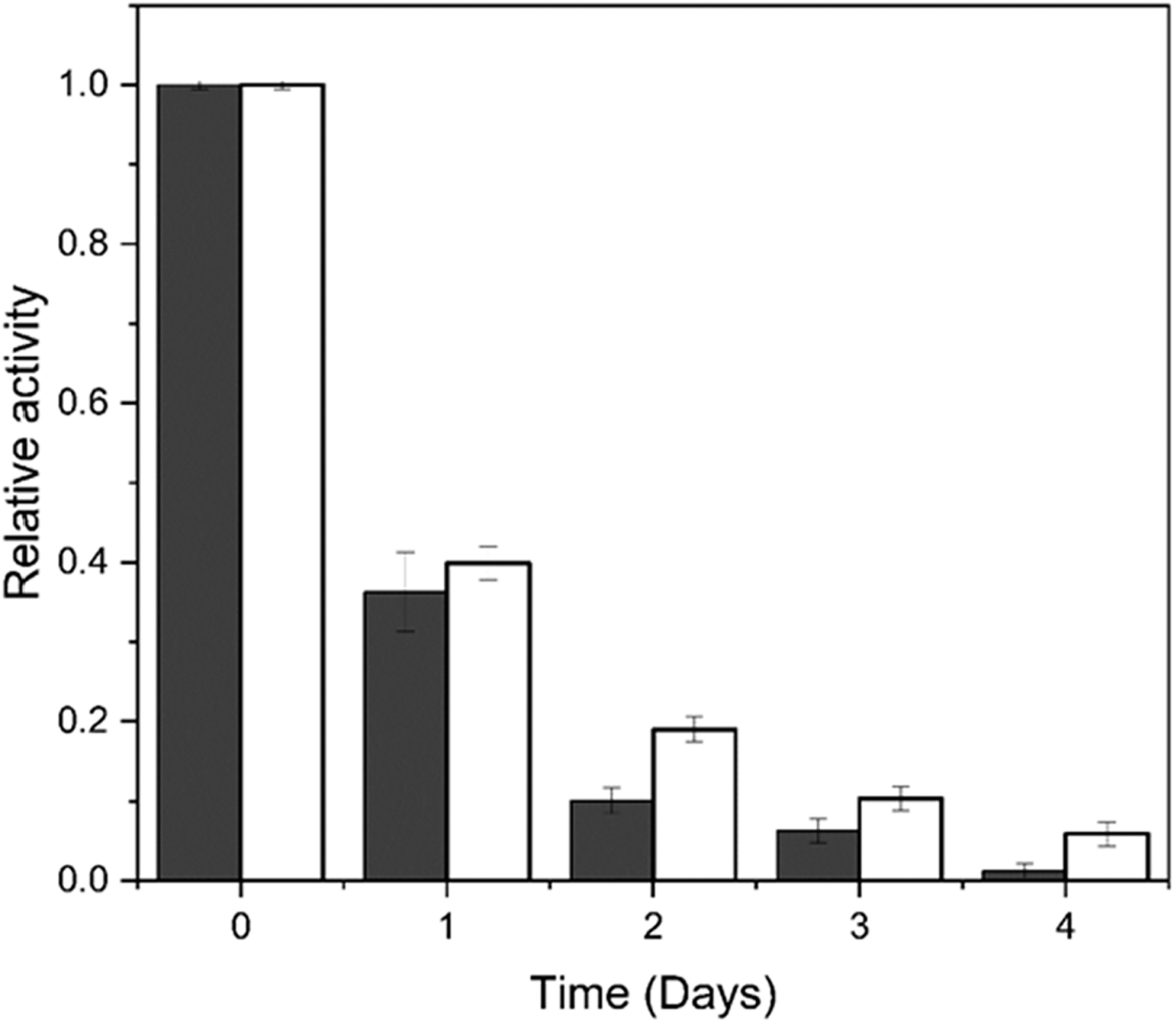
Storage stability of *A. oryzae* FTase immobilized onto onto silica gel (unfilled bar) and glutaraldehyde-functionalized silica gel (filled bar). Reaction conditions:: 47% (w v^–1^) sucrose solution and 0.2 mol L^–1^ of tris-acetate buffer (pH 5.5), 190 rpm at 50 °C for 60 min. The maximum activity for FTase immobilized onto functionalized silica gel (4.39 ± 0.05 U g^–1^), and FTase immobilized onto silica gel (3.22 ± 0.13 U g^–1^) were defined as 100% of relative activity.

A pronounced decrease in enzymatic activity was observed for both biocatalysts; nevertheless, the FTase immobilized on functionalized silica gel showed higher storage stability compared to the enzyme adsorbed on the unmodified silica gel. After 24 h of storage, the FTase immobilized on unmodified and functionalized silica gel retained 36.27% (1.1687 ± 0.0245 U g^–1^) and 39.89% (1.7497 ± 0.093U g^–1^) of their initial transfructosylation activities, respectively. Over the storage period, both systems exhibited a progressive decline in activity. Notably, after 96 h, the FTase immobilized on functionalized silica gel maintained 6% (0.2574 ± 0.0654 U g^–1^) of its initial activity, four times higher than that of the enzyme immobilized on unmodified silica gel. These results clearly demonstrate that glutaraldehyde functionalization of the inorganic support enhances FTase storage stability. Storage stability is a critical parameter in evaluating the industrial applicability of enzymes, since it contributes to reducing both processing time and operational costs.^50^ Therefore, the higher stability achieved by immobilization directly increases the feasibility of employing biocatalysts in large-scale industrial processes.^13,51^

### Operational Stability of the Immobilized Enzyme

Figure 8 shows the operational stability of the FTase immobilized onto silica gel and glutaraldehyde-functionalized silica gel over eight consecutive 1-h reaction cycles in a batch reactor. Both biocatalysts exhibited an initial decrease in activity after the first cycle.

**Figure 8:**
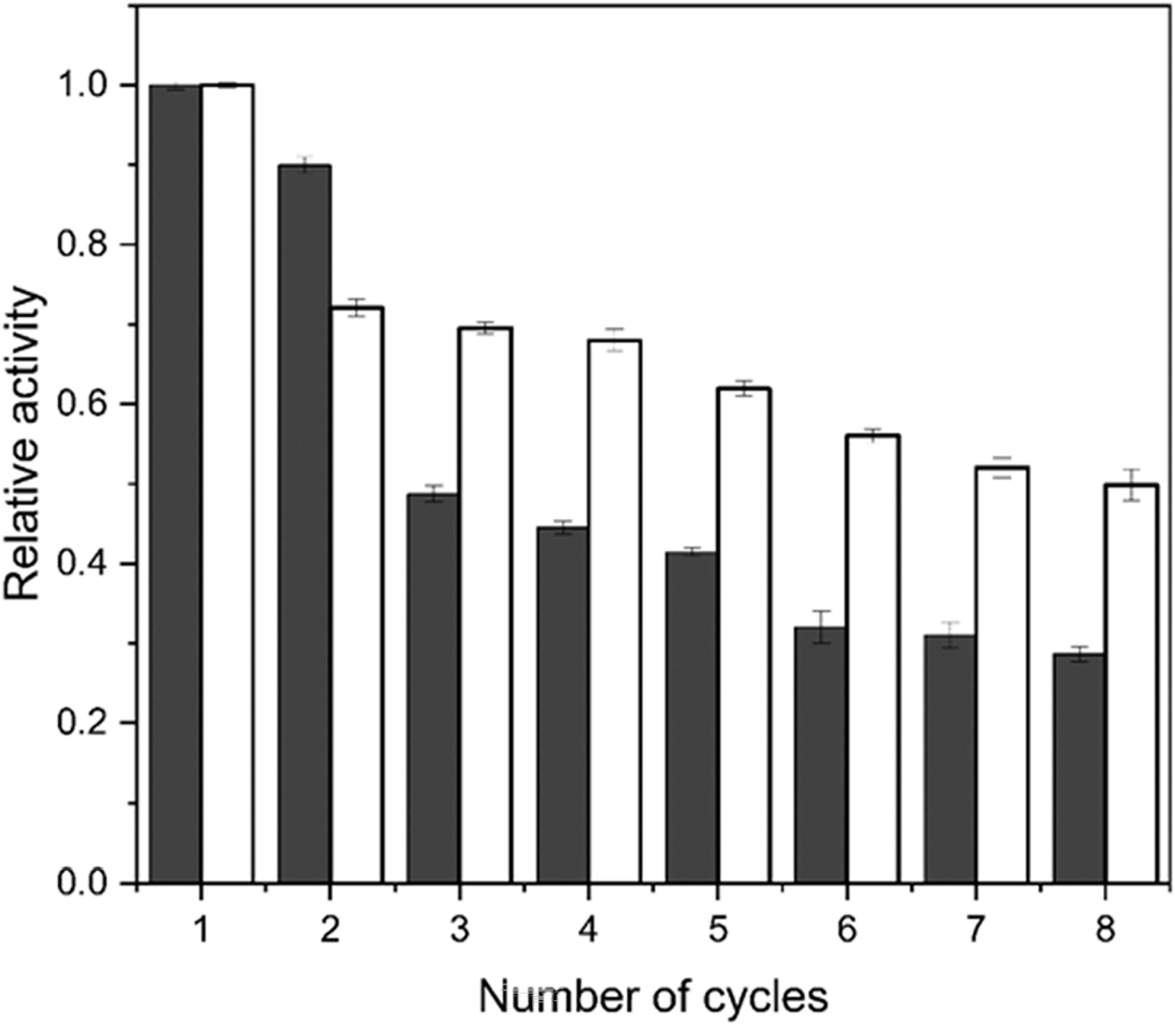
Operational stability of the FTase from *A. oryzae* IPT-301 immobilized onto silica gel (unfilled bar) and glutaraldehyde-functionalized silica gel (filled bar) during eight consecutive reaction batches. Reaction conditions: 47% (w v^–1^) sucrose solution and 0.2 mol L^–1^ of tris-acetate buffer (pH 5.5), 190 rpm at 50 °C for 60 min. The maximum activity for FTase immobilized onto functionalized silica gel (3.86 ± 0.16 U g^–1^), and FTase immobilized onto silica gel (3.57 ± 0.18 U g^–1^) were defined as 100% of relative activity.

The more pronounced loss observed after the second cycle suggests that superficial enzymes may have been removed from the support during filtration and washing, while enzymes embedded in deeper cavities remained bound.^2,52^ For the functionalized silica gel, the activity remained relatively stable from the second to the fourth cycle. By the end of the eighth cycle, the FTase immobilized on the functionalized support retained approximately 50% (1.8471 ± 0.0497 U g^–1^) of its initial activity, whereas the immobilization on the unmodified silica gel resulted in a residual activity of about 30% (1.4266 ± 0.0591 U g^–1^). The functionalization of the support with glutaraldehyde likely promoted the formation of stronger enzyme-support linkages, hindering enzyme desorption or denaturation and, thus, enhancing operational stability.^13,16^ In contrast, the lower stability of the FTase adsorbed onto the unmodified silica gel can be attributed to the weaker interactions characteristic of physical adsorption, including van der Waals forces, hydrogen bonding between silanol groups on the support and polar groups of the protein, and electrostatic interactions between negatively charged silanol groups (at pH 5.5) and positively charged regions of the enzyme.^2,13,16^

Sugahara and Varéa^52^ reported the reuse of an extracellular lipase from *Beauveria bassiana*, immobilized by adsorption on silica, over four reaction cycles, retaining 80.8% of its initial activity. Soares et al.^53^ demonstrated that *Candida rugosa* lipases immobilized by covalent bonding on high-porosity silica maintained their activity for up to 20 reuse cycles. An FTase from *A. oryzae* IPT-301 immobilized on polyhydroxybutyrate and glutaraldehyde-activated polyhydroxybutyrate retained more than 40% and 55% of the initial activity after six sequential reaction batches, respectively.^13^ It was also reported that the FTase from the same microorganism, immobilized onto silica gel^2^ and on alkali-treated corncob particles,^20^ retained 33% and 3.8% of its initial activity, respectively, after the sixth batch reaction cycle. Despite the activity losses observed for the FTase immobilized on both supports (Figure 8), the results suggest that the biocatalysts immobilized onto silica gel and, especially, on functionalized silica gel, hold great potential for reuse in batch FOS production, which could contribute to reducing production costs.^54^

## Conclusions

This study demonstrated that immobilizing FTase onto glutaraldehyde-functionalized silica gel represents an effective strategy for FOS production. Although the functionalized silica exhibited lower recovered activity compared to the unmodified silica gel, it provided higher immobilization yield and conferred enhanced thermal, operational, storage, and pH stability to the biocatalyst. The biocatalyst’s capacity for reuse over multiple reaction cycles further reinforces its potential for application in continuous or batch industrial processes. These results contribute to the advancement of heterogeneous biocatalytic systems, which can improve the economic and operational viability of large-scale enzymatic processes.

## Data Availability

All data supporting the findings of this study are included in the article.

## References

[1] Martínez D., Sobrino A., Aguiar A., González-Bacerio J., Hernández L., Pérez E. R., del Monte-Martínez A. (2024). Rational Design
and Immobilization of a Recombinant Sucrose:sucrose 1-Fructosyltransferase
on Sepabeads and ReliZyme Supports for Short-Chain Fructooligosaccharides
Production. Process Biochem..

[2] Faria L. L., Morales S. A. V., Prado J. P. Z., Dias G. S., Almeida A. F., Xavier M. C. A., Silva E. S., Maiorano A. E., Perna R. F. (2021). Biochemical
Characterization of Extracellular Fructosyltransferase from *Aspergillus oryzae* IPT-301 Immobilized on Silica
Gel for the Production of Fructooligosaccharides. Biotechnol. Lett..

[3] Cunha J. S., Ottoni C. A., Morales S. A. V., Silva E. S., Maiorano A. E., Perna R. F. (2019). Synthesis and Characterization
of Fructosyltransferase
from *Aspergillus oryzae* IPT-301 for
High Fructooligosaccharides Production. Braz.
J. Chem. Eng..

[4] Castro C. C., Nobre C., Duprez M. E., Weireld G., Hantson A. L. (2017). Screening
and Selection of Potential Carriers to Immobilize *Aureobasidium
pullulans* Cells for Fructo-Oligosaccharides Production. Biochem. Eng. J..

[5] Fernandez R. C., Ottoni C. A., Silva E. S., Matsubara R. S., Carter J. M., Magossi L. R., Wada M. A. A., Rodrigues M. F. A., Guilarte B. M., Maiorano A. E. (2007). Screening
of β-Fructofuranosidase
Producing Microorganisms and Effects of pH and Temperature on Enzymatic
Rate. Appl. Microbiol. Biotechnol..

[6] Yun J. W., Song S. K. (1996). Continuous Production of Fructooligosaccharides
Using
Fructofuranosidase Immobilized on Ion Exchange Resin. Biotechnol. Bioprocess Eng..

[7] Ghazi I., Fernandez-Arrojo L., De Segura A. G., Alcalde M., Plou F. J., Ballesteros A. (2006). Beet Sugar and Molasses as Low-Cost Feedstock for the
Enzymatic Production of Fructooligosaccharides. J. Agric. Food Chem..

[8] Platková Z., Polakovič M., Štefuca V., Vandálková M., Antošová M. (2006). Selection of Carrier for Immobilization
of Fructosyltransferase from *Aureobasidium pullulans*. Chem. Pap..

[9] Canilha L., Carvalho W., Silva J. B. A. (2006). Biotacalisadores
Imobilizados. Biotecnol. Cienc. Desenvolv..

[10] Souza, L. T. A. ; Veríssimo, L. A. A. ; Pessela, J. B. C. ; Santoro, M. M. ; Resende, R. R. ; Mendes, A. A. Enzyme Immobilization: Fundamental Principles and Support Types. In Biotecnologia Aplicada à Agro & Indústria; Blücher: São Paulo, 2017; pp 529–568 10.5151/9788521211150-15.

[11] Dias G. S., Santos E. D., Xavier M. C. A., Almeida A. F., Silva E. S., Maiorano A. E., Perna R. F., Morales S. A. V. (2022). Study on the
Transfructosylation Activity of *Aspergillus oryzae* IPT-301 Cells in a Packed Bed Reactor Aiming at Fructooligosaccharide
Production. J. Chem. Technol. Biotechnol..

[12] Oliveira E., Maugeri F. (2013). Effect of Sucrose and
Salts on FOS Synthesis. J. Food Biochem..

[13] Araújo I. M., Becalette P. C., Silva E. S., Dias G. S., Xavier M. C. A., Almeida A. F., Maiorano A. E., Morales S. A. V., Perna R. F. (2023). Enhancement
of Fructosyltransferase Stability by Immobilization on Polyhydroxybutyrate
and Glutaraldehyde-Activated Polyhydroxybutyrate for Fructooligosaccharides
Production. J. Chem. Technol. Biotechnol..

[14] Pereira A. C., Kubota L. T. (2004). Optimization of the Preparation of Carbon Paste Electrodes
Containing Riboflavin Immobilized on an Inorganic Support. Quím. Nova.

[15] Paula A. V., Urioste D., Santos J., De Castro H. (2007). Porcine Pancreatic
Lipase Immobilized on Polysiloxane–Polyvinyl Alcohol Hybrid
Matrix: Catalytic Properties and Feasibility to Mediate Synthesis
of Surfactants and Biodiesel. J. Chem. Technol.
Biotechnol..

[16] Mendes A.
A., De Castro H. F., Giordano R. L. C. (2013). Screening of Organic Supports and
Activation Protocols for the Immobilization and Stabilization of Lipase
from *Thermomyces lanuginosus*. Quím. Nova.

[17] Barbosa O., Torres R., Ortiz C., Fernandez R. (2012). Versatility
of Glutaraldehyde to Immobilize Lipases: Effect of the Immobilization
Protocol on the Properties of Lipase B from *Candida
antarctica*. Process Biochem..

[18] Gonçalves M. C.
P., Morales S. A. V., Silva E. S., Maiorano A. E., Perna R. F., Kieckbusch T. G. (2020). Entrapment
of Glutaraldehyde-Crosslinked
Cells from *Aspergillus oryzae* IPT-301
in Calcium Alginate for High Transfructosylation Activity. J. Chem. Technol. Biotechnol..

[19] Silva M. B. P. O., Abdal D., Prado J. P. Z., Dias G. S., Morales S. A. V., Xavier M. C. A., Almeida A. F., Silva E. S., Maiorano A. E., Perna R. F. (2021). Effect of Temperature, pH and Storage Time on the Stability
of an Extracellular Fructosyltransferase from *Aspergillus
oryzae* IPT-301. Braz. J. Food
Technol..

[20] Pereira R. S., Vieira A. C., Leite P. C., Maestrelli S. C., Silva E. S., Maiorano A. E., Xavier M. C. A., Lopes M. S., de Paula A. V., Morales S. A. V., Perna R. F. (2025). Application of an
Agro-Waste for the Immobilization of Microbial Fructosyltransferase:
A New Alternative for Fructooligosaccharide Production. J. Braz. Chem. Soc..

[21] Brunauer S., Emmett P. H., Teller E. (1938). Adsorption of Gases in Multimolecular
Layers. J. Am. Chem. Soc..

[22] Barrett E. P., Joyner L. G., Halenda P. P. (1951). The Determination
of Pore Volume
and Area Distributions in Porous Substances. I. Computations from
Nitrogen Desorption Isotherms. J. Am. Chem.
Soc..

[23] Garcia R. L., Dias G. S., Morales S. A. V., Xavier M. C. A., Silva E. S., Maiorano A. E., Tardioli P. W., Perna R. F. (2021). Glutaraldehyde-Crosslinked
Cells from *Aspergillus oryzae* IPT-301
for High Transfructosylation Activity: Optimization of the Immobilization
Variables, Characterization and Operational Stability. Braz. J. Chem. Eng..

[24] Sadana A., Henley J. P. (1987). Single-Step Unimolecular Non-First-Order
Enzyme Deactivation
Kinetics. Biotechnol. Bioeng..

[25] Blanco E. M., Horton M. A., Mesquida P. (2008). Simultaneous
Investigation of the
Influence of Topography and Charge on Protein Adsorption Using Artificial
Nanopatterns. Langmuir.

[26] Barthee R., Vijila K. (2014). Study on Fructosyltransferase
Enzyme from Aspergillus
sp. in Fructooligosaccharides Production. Res.
J. Recent Sci..

[27] Juszczak L., Fortuna T., Wodnicka K. (2002). Characteristics
of Cereal Starch
Granules Surface Using Nitrogen Adsorption. J. Food Eng..

[28] Gomes L. S., Furtado A. C. R., Souza M. C. (2018). The Silica and Its Particularities. Rev. Virtual Quím..

[29] An D., Guo Y., Zhu Y., Wang Z. (2010). A Green Route to Preparation
of Silica
Powders with Rice Husk and Waste Gas. Chem.
Eng. J..

[30] Gao G.-M., Zou H., Gan S., Liu Z., An B., Xu J., Li G. (2009). Preparation
and Properties of Silica Nanoparticles from Oil Shale
Ash. Powder Technol..

[31] Liou T. H., Lin H. S. S. (2012). Synthesis and
Surface Characterization of Silica Nanoparticles
from Industrial Resin Waste Controlled by Optimal Gelation Conditions. J. Ind. Eng. Chem..

[32] Socrates, G. Infrared and Raman Characteristic Group Frequencies: Tables and Charts, 3rd ed.; Wiley: New York, 2001.

[33] Mishra S., Hansda B., Ghosh A., Mondal S., Mandal B., Kumari P., Das B., Mondal T. K., Biswas T. (2023). Multipoint
Immobilization at Inert Center of Papain on Homo-Functional Diazo-Activated
Silica Support: A Way of Restoring “Above Room-Temperature”
Bio-Catalytic Sustainability. Langmuir.

[34] Zhai T., Wang C., Gu F., Meng Z.-h., Liu W., Wang Y. (2020). Dopamine/Polyethylenimine-Modified
Silica for Enzyme Immobilization
and Strengthening of Enzymatic CO_2_ Conversion. ACS Sustainable Chem. Eng..

[35] Ren Y., Zhang L., Sun T., Yin Y., Wang Q. (2022). Enzyme Immobilization
on a Delignified Bamboo Scaffold as a Green Hierarchical Bioreactor. ACS Sustainable Chem. Eng..

[36] Datta S., Christena L. R., Rajaram Y. R. S. (2013). Enzyme immobilization: an overview
on techniques and support materials. 3Biotech.

[37] Carvalho N. B., Lima A. S., Soares C. M. F. (2014). Use
of Modified Silicas for Lipase
Immobilization. Quím. Nova.

[38] Erdemir S., Yilmaz M. (2009). Synthesis of Calix­[n]­arene-Based
Silica Polymers for
Lipase Immobilization. J. Mol. Catal. B: Enzym..

[39] Vescovi V., Giordano R. L. C., Mendes A. A., Tardioli P. W. (2017). Immobilized Lipases
on Functionalized Silica Particles as Potential Biocatalysts for the
Synthesis of Fructose Oleate in an Organic Solvent/Water System. Molecules.

[40] Ferreira M. M., Santiago F. L. B., Da Silva N. A. G., Luiz J. H. H., Fernández-Lafuente R., Mendes A. A., Hirata D. B. (2018). Different
Strategies to Immobilize
Lipase from *Geotrichum candidum*: Kinetic
and Thermodynamic Studies. Process Biochem..

[41] Garcia L. A., Prado P. J. Z., Morales S. A. V., Silva E. S., Maiorano A. E., Xavier M. C. A., Lopes M. S., Gunnewiek R. F. K., Perna R. F. (2022). A New Support for Fructosyltransferase
(FTase, E.C.
2.4.1.9) Immobilization Based on Niobia-Silica Mixed Oxide for Fructooligosaccharides
Production. Mater. Today Commun..

[42] Alvarado-Huallanco M. B., Maugeri-Filho F. (2010). Kinetics and
Modeling of Fructo-Oligosaccharide Synthesis
by Immobilized Fructosyltransferase from *Rhodotorula sp*. J. Chem. Technol. Biotechnol..

[43] da
Costa Luchiari I., Cedeno F. R. P., Farias T. A. M., Picheli F. P., de Paula A. V., Monti R., Masarin F. (2021). Glucoamylase Immobilization
in Corncob Powder: Assessment of Enzymatic Hydrolysis of Starch in
the Production of Glucose. Waste Biomass Valor.

[44] Mateo E. M., Palomo J. M., Fernandez-Lorente G., Guisan J. M., Fernandez-Lafuente R. (2007). Improvement
of Enzyme Activity, Stability and Selectivity via Immobilization Techniques. Enzyme Microb. Technol..

[45] Saqib A. A. N., Hassan M., Khan N. F., Baig S. (2010). Thermostability of
Crude Endoglucanase from *Aspergillus fumigatus* Grown under Solid State Fermentation (SSF) and Submerged Fermentation
(SmF). Process Biochem..

[46] Kitahara H., Nakamura T., Ito T., Kajiwara Y., Nakajima M. (1997). Purification
and Characterization of a Fructosyltransferase from *Aspergillus oryzae* K.B. F-2029. Process Biochem..

[47] Coelho A. V., Lima M. S., Oliveira R. S., Teixeira J. A., Queiroz J. A., Azevedo A. M. (2017). Immobilization of a Thermostable
α-Amylase on
Chitosan Beads for Starch Hydrolysis. Molecules.

[48] Bezbradica D. I., Mateo J. M., Guisan J. (2014). Novel Support
for Enzyme Immobilization
Prepared by Chemical Activation with Cysteine and Glutaraldehyde. J. Mol. Catal. B: Enzym..

[49] Xu Q., Zheng X., Huang M., Wu M., Yan Y., Pan J., Yang Q., Duan C. J., Liu J. L., Feng J. X. (2015). Purification
and biochemical characterization of a novel fructofuranosidase from *Penicillium oxalicum* with transfructosylating activity
producing neokestose. Process Biochem..

[50] Horn S. J., Sørlie M., Vaaje-Kolstad G., Norberg A. L., Synstad B., Vårum K. M., Eijsink V. G. H. (2005). Comparative Studies of Chitinases
A, B and C from *Serratia marcescens*. FEBS J..

[51] Perna R. F., Miranda E., Silva M. C., Maiorano A. E., Bon E. P. S., Pastore G. M., Gunnewiek R. F. K., Giordano R. L. C., de
Castro R. A. (2019). Production of Fructooligosaccharides from Sucrose by
a Novel Fructosyltransferase from *Aspergillus oryzae* and Its Prebiotic Potential. Sci. Rep..

[52] Sugahara V. H., Varéa G. S. (2014). Immobilization
of *Beauveria bassiana* Lipase by Adsorption
on Silica Gel for Enhanced Activity and Stability
in Aqueous and Organic Media. Braz. Arch. Biol.
Technol..

[53] Soares C. M. F., De Castro H. F., De Moraes F. F., Zanin G. M. (1999). Characterization
and Utilization of *Candida rugosa* Lipase
Immobilized on Controlled Pore Silica. Appl.
Biochem. Biotechnol..

[54] Guisán, J. M. Biocatalysis and Industrial Applications. In Advances in Enzyme Immobilization Techniques; Springer: New York, 2018; pp 15–32.

